# Intratumoral and Peritumoral Functional Connectivity Reveals Network‐Level Signatures of Glioma

**DOI:** 10.1002/mco2.71083

**Published:** 2026-10-17

**Authors:** Hongbo Bao, Yan Bai, Jie Hu, Wencai Ding, Jiacheng Lai, Andrew Zalesky, Ye Ella Tian, Yanmei Tie, Janet Mei, Shengyu Fang, Xing Fan, Licia P. Luna, Craig K. Jones, Jianxun Ren, Weigang Cui, Jiadong Yan, Zeya Yan, Zihan Wang, Renwu Zhang, Zhonghua Lv, Yihang Jiao, Tao Jiang, Peng Liang, Haris I. Sair, Yinyan Wang

**Affiliations:** ^1^ Department of Neurosurgery Beijing Tiantan Hospital Capital Medical University Beijing China; ^2^ Beijing Neurosurgical Institute Capital Medical University Beijing China; ^3^ Department of Neurosurgery Harbin Medical University Cancer Hospital Harbin China; ^4^ The Russell. H. Morgan Department of Radiology and Radiological Science Johns Hopkins University School of Medicine Baltimore USA; ^5^ Department of Laboratory Construction and Equipment Management Heilongjiang University Harbin China; ^6^ Department of Neurology The Second Affiliated Hospital of Wannan Medical College Wuhu China; ^7^ Systems Group Department of Psychiatry The University of Melbourne Melbourne Australia; ^8^ Department of Neurosurgery Harvard Medical School Brigham and Women's Hospital Boston USA; ^9^ Department of Computer Science Johns Hopkins University Baltimore USA; ^10^ The Malone Center for Engineering in Healthcare The Whiting School of Engineering Johns Hopkins University Baltimore USA; ^11^ Division of Brain Sciences Changping Laboratory Beijing China; ^12^ McGill Centre for Integrative Neuroscience McGill University Montreal Canada; ^13^ Department of Neurological Surgery Johns Hopkins University School of Medicine Baltimore USA

**Keywords:** fMRI, functional network, glioma, intratumoral functional region

## Abstract

Gliomas are recognized as network‐level diseases that remodel functional connectivity across the brain. Using resting‐state functional MRI (rs‐fMRI) data from 306 patients with pathologically confirmed glioma at two hospitals, we delineated individualized intra‐ and peritumoral functional networks to quantify tumor‐related alterations. Two novel indices—the ratio of intratumoral functional regions (RIFR) and ratio of peritumoral tumor‐related regions (RPTR)—were proposed to describe network remodeling. Both indices were related to pathological, structural, functional, cognitive, language, and survival measures. Lower RIFR indicated diminished intratumoral connectivity and worse prognosis, while higher values reflected preserved network integration and longer survival. Models incorporating network features yielded areas under the receiver operating characteristic curve of 0.90 in training and 0.86–0.88 in external validation. Supporting analyses suggested that intratumoral functional regions are associated with distinct molecular and cellular characteristics. These findings suggest that intra‐ and peritumoral networks reflect biologically and functionally distinct tumor subregions and are associated with network‐level alterations, brain function, and patient outcome. Interpretation is limited by the hemodynamic basis of rs‐fMRI, incomplete data, and limited intraoperative validation. These findings identify individualized intra‐ and peritumoral connectivity measures as candidate markers of glioma‐associated network remodeling and prognosis that require prospective, multimodal validation.

## Graphical abstract

Using resting‐state fMRI in 306 glioma patients, this study maps individualized intra‐ and peritumoral functional networks. Novel metrics (RIFR, RPTR) quantify tumor‐related network remodeling, correlating with pathology, structure, cognition, and survival. A prognostic model incorporating these network features achieves an AUC of 0.90. These findings suggest network‐based measures as candidate prognostic markers warranting further validation.

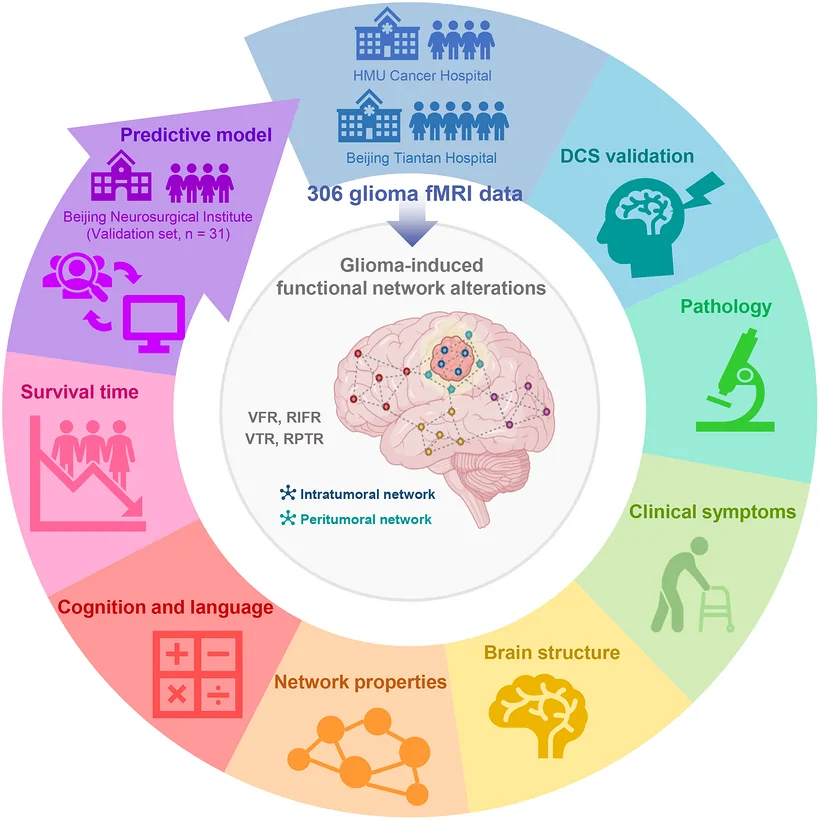

## 1 Introduction

Gliomas, the most common type of primary malignant brain tumor, not only disrupt local cellular environments but also influence global brain network functionality [1, 2]. The burgeoning field of cancer neuroscience has highlighted the complex interplay between tumor development and neural network alterations across multiple scales of brain organization [3]. At a microscopic or cellular level, gliomas interact with neuronal synapses, promoting tumor growth and progression through mechanisms akin to neurodevelopmental processes [4]. Mesoscopically, gliomas integrate into neural circuits, utilizing neurotransmitter systems to further their invasive behavior [4, 5, 6]. These observations challenge a lesion‐centered view of glioma and motivate assessment of the tumor as part of the neural environment.

At the macroscale, gliomas are associated with altered functional connectivity across distributed brain systems, which may contribute to epilepsy and cognitive, language, and motor impairment [7, 8]. Functional magnetic resonance imaging (fMRI), particularly resting‐state fMRI (rs‐fMRI), provides a noninvasive means to characterize these changes without requiring task performance [3, 9]. Prior studies have described local peritumoral remodeling and widespread network disruption, indicating that clinically relevant effects may arise from both the lesion and its interactions with the remaining brain [10, 11].

Functional connectivity has also been detected within the tumor mass, suggesting that some intratumoral regions remain coupled to established brain networks [12]. Although glioma cells themselves do not produce classical neuronal electrophysiological signals [13], the fMRI‐derived functional connectivity likely reflects indirect neurovascular or metabolic interactions, as well as potential coupling with infiltrated neurons or tumor–neuron communication pathways. These findings are consistent with cellular‐level evidence demonstrating that glioma cells can incorporate into neuronal circuits [14, 15]. Existing evidence supports the biological plausibility of tumor integration into neural circuits, but the relationships of individualized intratumoral and peritumoral connectivity with tumor biology, whole‐brain organization, neurological function, and prognosis remain insufficiently characterized.

This study aimed to delineate individualized intratumoral and peritumoral functional networks in a two‐center glioma cohort and to evaluate their associations with pathological and molecular features, brain structure and network topology, cognitive and language function, and survival. We hypothesized that the extent of preserved intratumoral connectivity and peritumoral coupling would differ across glioma phenotypes and would be associated with clinically relevant network disruption and outcomes. By jointly characterizing tissue within and around the tumor, this framework was intended to provide a patient‐specific view of glioma‐associated network remodeling and to support evaluation of connectome‐derived measures as candidate imaging biomarkers.

## 2 Results

### 2.1 Mapping of Intratumoral and Peritumoral Functional Networks

In this study, a total of 306 glioma patients (124 female/182 male, age: 44.08 ± 11.79 years) from two centers were included for further analyses (Figure 1). Patients’ demographic, pathological, and clinical characteristics are detailed in Table 1 and Figure S1. We maintained the delineation of 17 brain networks in the normal brain tissue surrounding the tumors and considered the core tumor as a separate, individual tumor network. This approach allowed us to recalibrate the network affiliations for each voxel within the core tumor and peritumoral signal abnormality (PSA) areas, identifying 17+1 distinct brain networks at an individual level (Figure 2).

**FIGURE 1:**
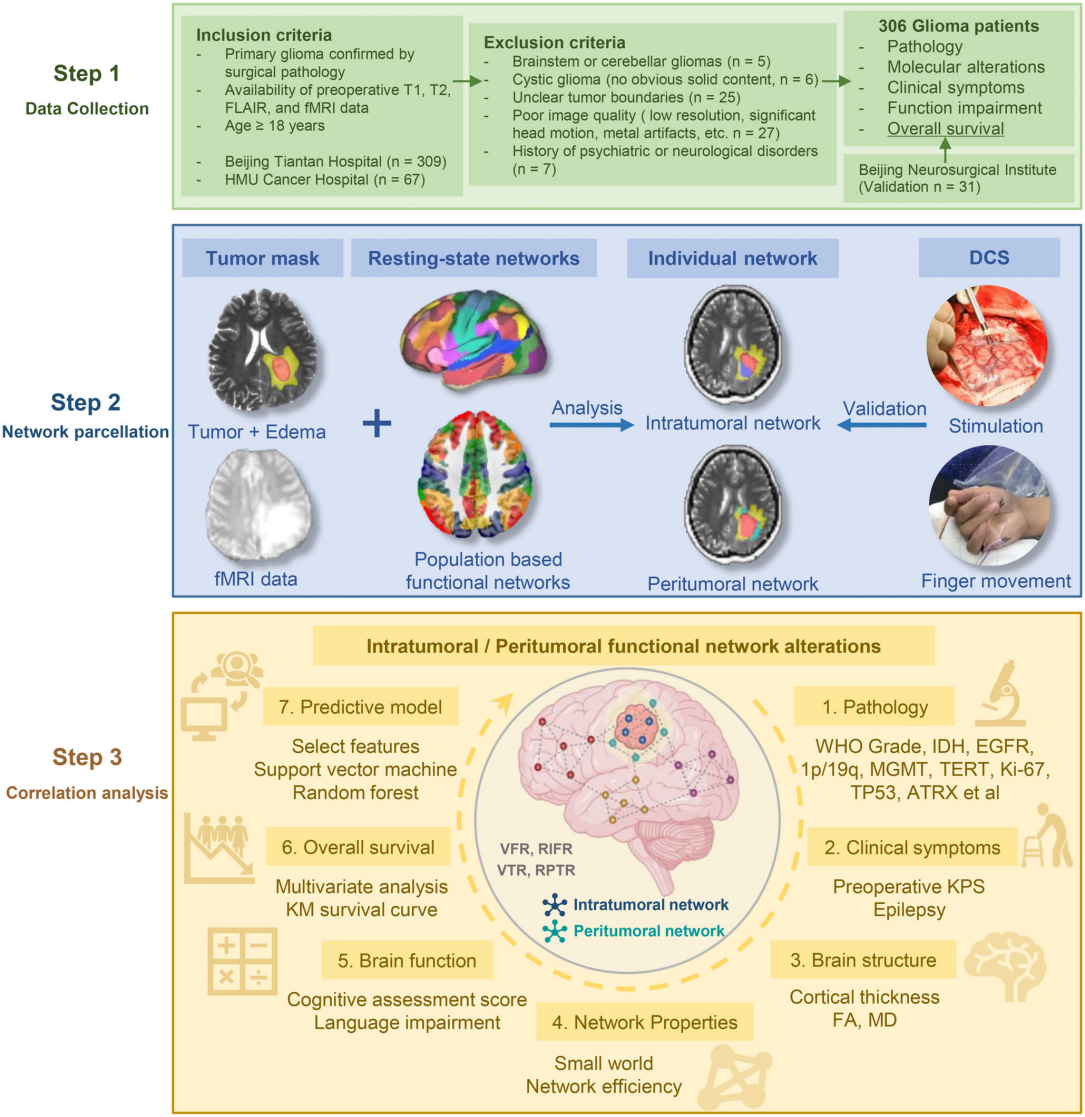
Flow diagram of study design.

**TABLE 1:** Baseline characteristics of the study sample.

Characteristics	*n* = 306
**Sex, *n* (%)**	
Female	124 (40.5%)
Male	182 (59.5%)
**Age (years)**	
Median (IQR)	44 (35, 52.75)
Mean ± SD	44.08 ± 11.79
**WHO grade, *n* (%)**	
Grade I	3 (1.0%)
Grade II	163 (53.3%)
Grade III	74 (24.2%)
Grade IV	66 (21.6%)
**Molecular subtypes, *n* (%)**	
GBM	69 (22.5%)
Astrocytoma	82 (26.8%)
Oligodendroglioma	64 (20.9%)
Unknown	91 (29.7%)
**IDH, *n* (%)**	
Mutation	148 (48.4%)
Wildtype	74 (24.2%)
Unknown	84 (27.5%)
**EGFR, *n* (%)**	
Positive	64 (20.9%)
Negative	60 (19.6%)
Unknown	182 (59.5%)
**1p/19q, *n* (%)**	
1p/19q codel	69 (22.5%)
1p/19q noncodel	142 (46.4%)
Unknown	95 (31.0%)
**Ki‐67, *n* (%)**	
Positive	179 (58.5%)
Negative	86 (28.1%)
Unknown	41 (13.4%)
**TP53, *n* (%)**	
Mutation	25 (8.2%)
Wildtype	40 (13.1%)
Unknown	241 (78.8%)
**MGMT promoter, *n* (%)**	
MGMT met	174 (56.9%)
MGMT unmet	56 (18.3%)
Unknown	76 (24.8%)
**TERTp, *n* (%)**	
Mutation	136 (44.4%)
Wildtype	33 (10.8%)
Unknown	137 (44.8%)
**ATRX, *n* (%)**	
Positive	129 (42.2%)
Negative	111 (36.3%)
Unknown	66 (21.6%)
**KPS, *n* (%)**	
Mean ± SD	84.69 ± 8.31
≥90	82 (26.8%)
<90	110 (35.9%)
Unknown	114 (37.3%)
**Epilepsy, *n* (%)**	
With epilepsy	84 (27.5%)
Without epilepsy	109 (35.6%)
Unknown	113 (36.9%)
**Cognition, *n* (%)**	
Normal	43 (14.1%)
Mild	21 (6.9%)
Moderate to severe	15 (4.9%)
Unknown	227 (74.2%)
**Language, *n* (%)**	
Normal	68 (22.2%)
Impaired	17 (5.6%)
Unknown	221 (72.2%)

Abbreviations: ATRX, alpha thalassemia/mental retardation syndrome X‐linked; EGFR, epidermal growth factor receptor; GBM, glioblastoma; IDH, isocitrate dehydrogenase; IQR, interquartile range; KPS, Karnofsky Performance Status; MGMT, O6‐methylguanine‐DNA methyltransferase; SD, standard deviation; TERTp, telomerase reverse transcriptase promoter; WHO, World Health Organization.

**FIGURE 2:**
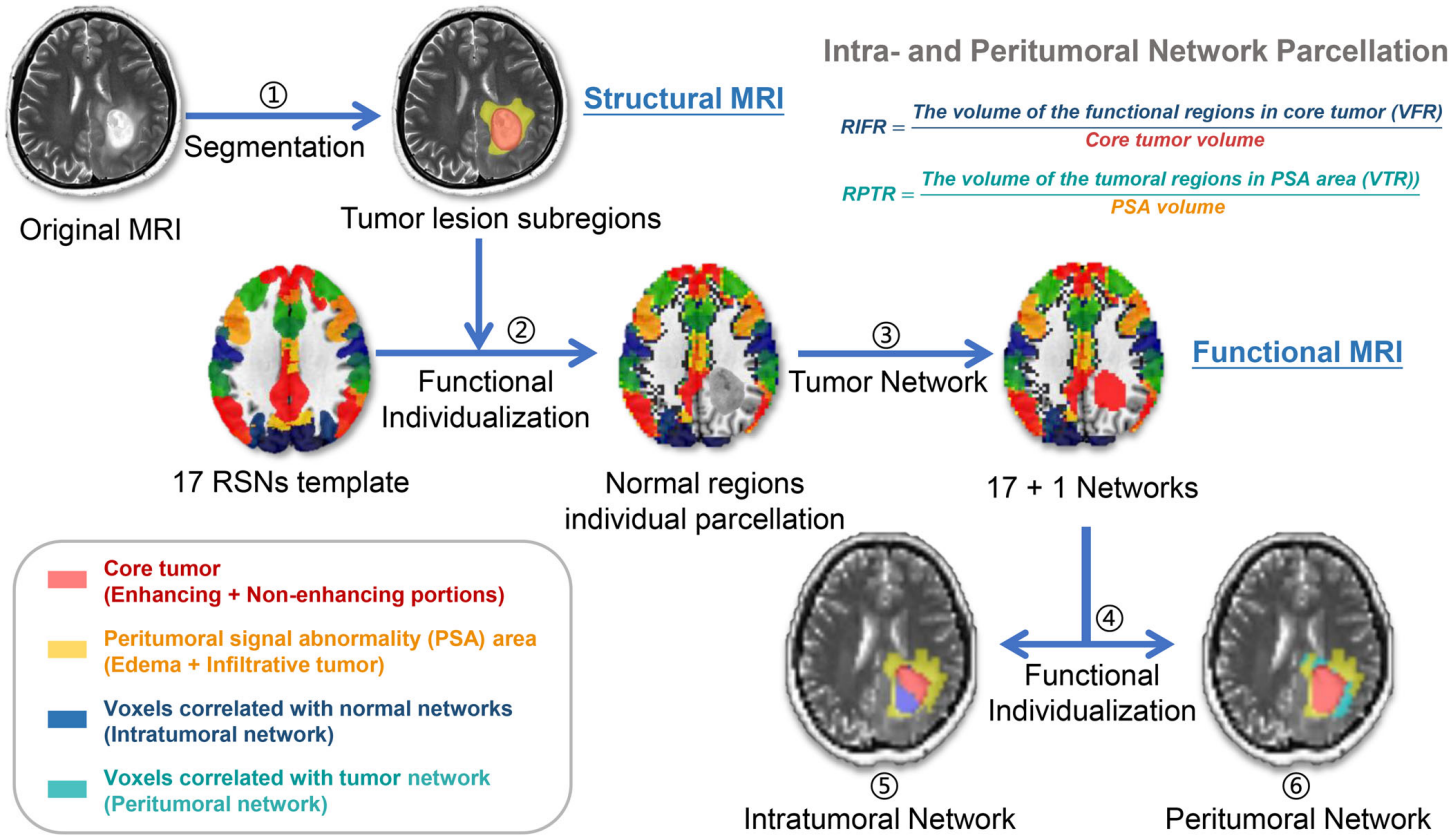
Schematic representation of the simplified individualized functional connectivity analysis for glioma patients. The process includes six main steps: (1) Segmentation of the tumor into the core tumor portion and PSA area, with external brain regions categorized as NAB. (2) Application of the Yeo 17 resting‐state networks (RSNs) atlas to parcellate the NAB regions. (3) Regard the core tumor portion as an independent network, combining it with the 17 normal RSNs to form a total of 18 functional networks within the brain. (4) Calculation of functional connectivity (FC) using the Spearman correlation coefficient, setting a threshold to define connectivity. (5) Identification of intratumoral functional regions by evaluating FC values above the threshold in comparison to the normal brain networks, calculating the volume and ratio of these regions (VFR and RIFR). (6) Identify PSA area voxels exceeding the FC threshold when compared with the tumor network, calculating the volume and ratio of these regions (VTR and RPTR). The FC threshold, set at 0.7 for optimal accuracy, was validated across a range from 0.3 to 0.7, with results from other thresholds detailed in the Supporting Information Materials. Voxels in which FC with any of the 17 canonical RSNs or tumor network exceeded the FC threshold were operationally defined as “functionally connected”. PSA, peritumoral signal abnormality; NAB, normal‐appearing brain; RSN, resting‐state network; FC, functional connectivity; VFR, volume of the functional regions in core tumors; RIFR, ratio of intratumoral functional regions to core tumor volume; VTR, volume of peritumoral tumor‐related regions; RPTR, ratio of peritumoral tumor‐related regions to PSA volume.

Quantitative assessments were conducted to describe the glioma‐related functional network features. This involved calculating the volume of the functional regions (VFR) in core tumors, the ratio of the intratumoral functional regions (RIFR) to core tumor volume, the volume of the tumoral regions (VTR) functionally related to the tumor network in the PSA area, and the ratio of the peritumoral tumoral regions (RPTR) to PSA volume. The results revealed that intratumoral regions exhibiting functional connectivity are prevalent across various molecular subtypes of gliomas (Figure 3A). In most cases, intratumoral functional regions were detected based on a functional connectivity (FC) threshold of 0.7, indicating strong correlations with one or more of the 17 canonical networks. Quantitatively, most core tumors, including both enhancing and nonenhancing portions, contained a small proportion of these functional areas: half of the tumors had 0% to 20% functional regions, while those with functional areas exceeding 80% were rare, accounting for only 1.63% of cases (Figure 3B). The distribution of intratumoral functional areas across different resting‐state networks (RSNs) varied, with higher proportions associated with the somatomotor, dorsal attention, salience/ventral attention, and default networks (Figure 3C). In addition, we found that the closer a tumor is to a brain region that is associated with a specific network, the higher its intratumoral RIFR associated with that network (Supporting Information Methods, Figures S2 and S3). For gliomas with heterogeneous enhancement (*n* = 27), further analysis compared the proportion of functional areas within solid enhancing tumor and nonenhancing necrotic core. Results indicated that the functional area proportion was significantly higher in enhancing regions compared with nonenhancing regions (Figure 3D, 0.142 ± 0.116 vs. 0.048 ± 0.064, *p* < 0.001). In 55.6% (15/27) of cases with enhancing tumors, the nonenhancing portion contained no functional regions (RIFR = 0).

**FIGURE 3:**
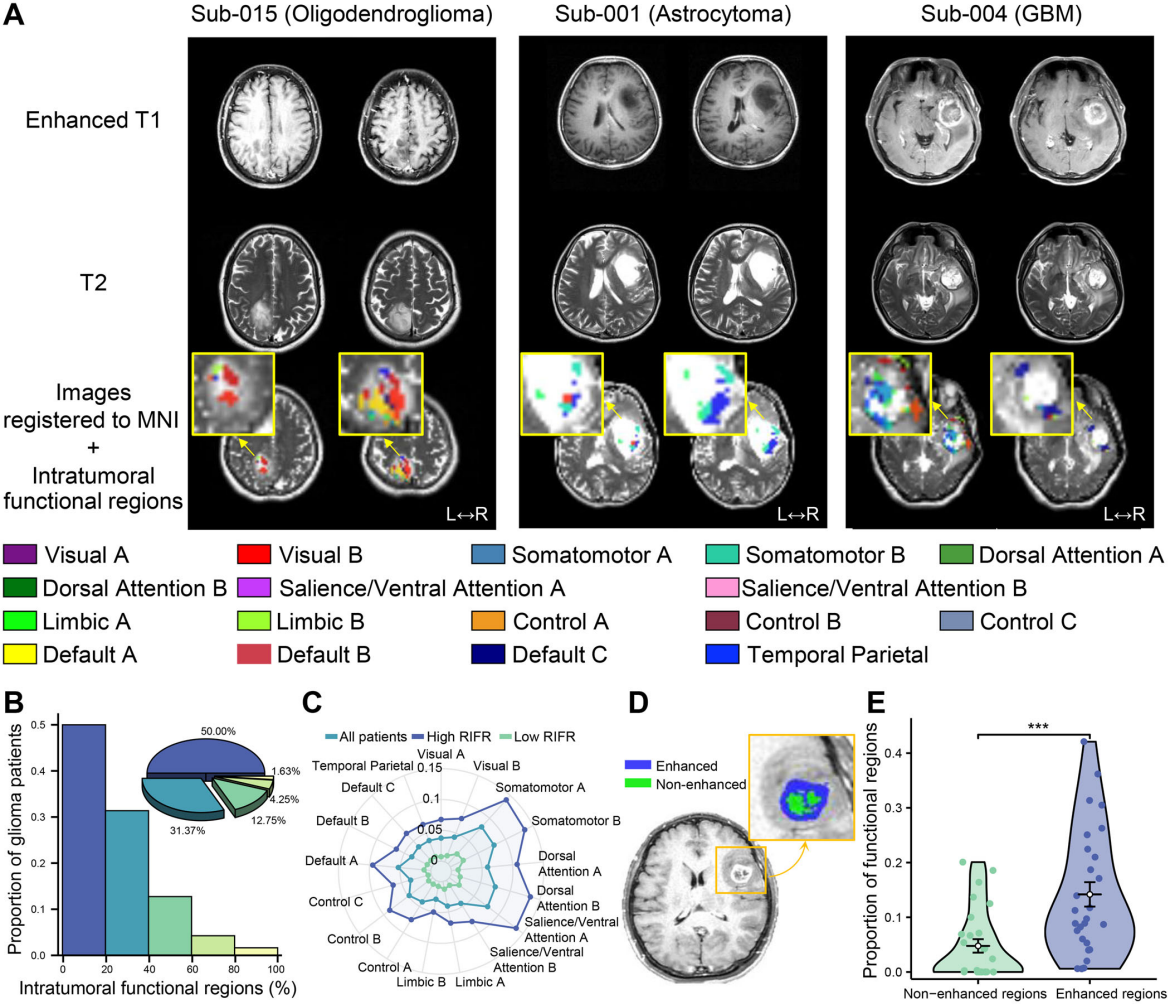
Individual parcellation of functional networks in glioma. (A) The delineation of individual‐specific intratumoral functional networks using an individual parcellation method across three representative glioma molecular subtypes in patients Sub‐015, Sub‐001, and Sub‐004. Each patient's series includes enhanced T1‐weighted images (upper panels), T2‐weighted images (middle panels), and their individual‐specific networks overlaid onto MRI images registered to the MNI space (bottom panels). Enlarged images within the yellow frames highlight the tumor lesions. The 17 functional networks are represented using a consistent color‐coding scheme detailed in the legend. (B) A histogram displaying the distribution of the percentage of intratumoral functional regions across glioma patients. (C) A radar chart showing the proportion of intratumoral functional areas corresponding to each of the 17 functional networks, with axis labels indicating the percentages. (D) Violin plots compare the proportion of functional regions within nonenhancing and enhancing areas of gliomas with ring enhancement (*n* = 27). Statistical significance is denoted by ^***^ *p* < 0.001. MNI, Montreal Neurological Institute.

### 2.2 Validation of Intratumoral Functional Areas by Direct Cortical Stimulation (DCS)

DCS is recognized as the current benchmark for intraoperative functional mapping [16], providing precise delineation of sensorimotor and language regions. In the primary retrospective cohort, seven patients had tumors in the region of the sensorimotor areas and underwent DCS during awake craniotomy (Figure S4). The functional territories within the tumor lesions delineated by our resting‐state fMRI (rs‐fMRI) method were aligned with the sensorimotor regions localized by DCS in six out of seven cases (85.7%; Figure 4). This high concordance supports the accuracy of our functional parcellation approach in detecting functional networks at the individual level.

**FIGURE 4:**
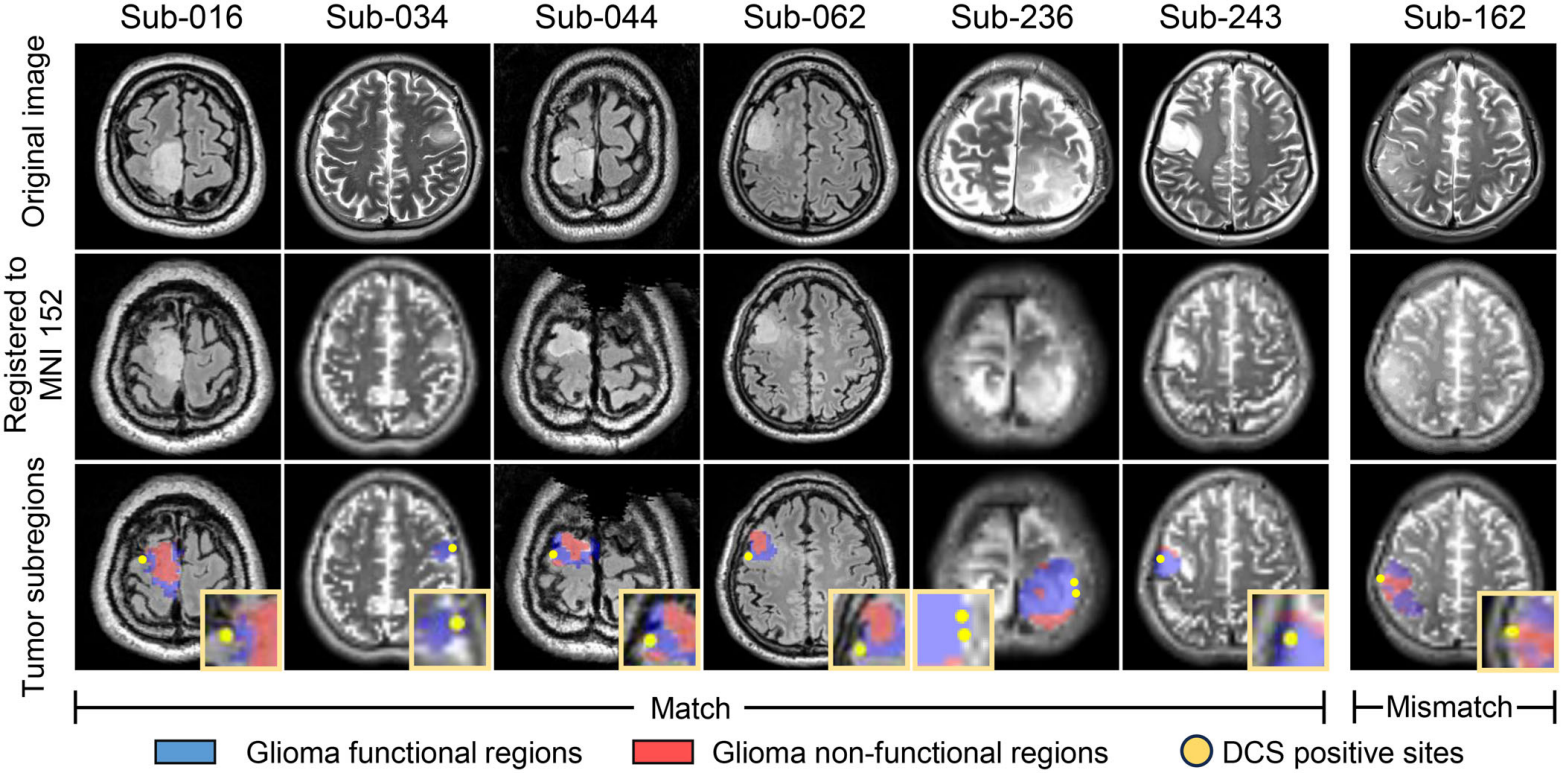
Overlap of DCS‐positive sites with intratumoral functional regions. The first row displays the original tumor lesion MRI images, while the second row shows the images after registration to the MNI space. The third row illustrates the intratumoral functional regions (blue), intratumoral nonfunctional regions (red), and the sensorimotor areas identified as DCS‐positive sites, demonstrating the spatial correlation and precision of functional mapping in the context of tumor‐induced brain network alterations. DCS, direct cortical stimulation.

### 2.3 Correlations of Individualized Brain Network Features With Pathology and Symptoms

To explore whether the functional regions within tumors are associated with pathological characteristics, clinical symptoms, and their impact on global brain network features and brain function, we conducted a multifaceted analysis. Initially, we compared the proportions of intratumoral functional areas across glioma subtypes with different grades and molecular characteristics. The results indicated that Grade IV, isocitrate dehydrogenase (IDH) wildtype, and epidermal growth factor receptor (EGFR)‐positive gliomas exhibited significantly lower RIFR compared with Grade I to III, IDH‐mutant, and EGFR‐negative tumors, respectively (Figure 5A–C). This suggests that higher malignancy gliomas tend to have lower RIFR. However, variables such as 1p/19q codeletion status, Ki‐67 positivity, TP53 mutation, O‐6‐methylguanine‐DNA methyltransferase (MGMT) promoter methylation, telomerase reverse transcriptase (TERT) promoter mutation, and alpha thalassemia/mental retardation syndrome X‐linked (ATRX) mutation did not influence RIFR (Figures 5D; Figure S5).

**FIGURE 5:**
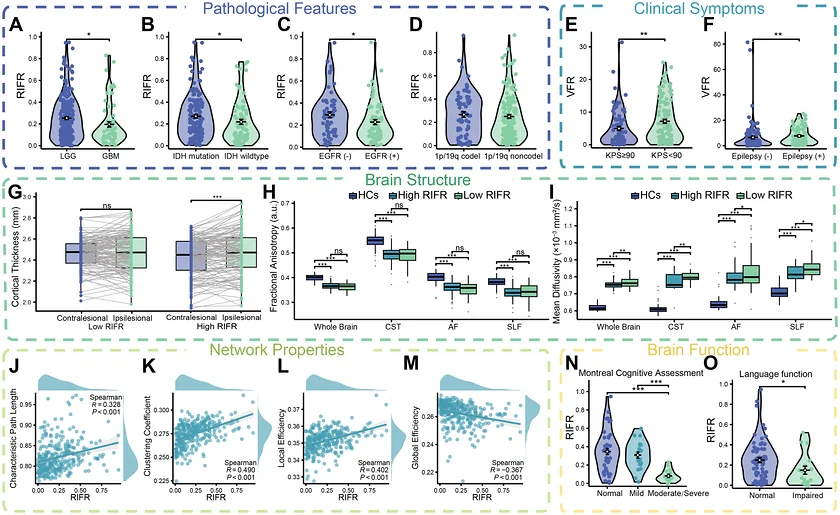
Correlations between intratumoral functional regions and various pathological features, clinical manifestations, structure, network properties, and brain function impairments. (A–D) Violin plots illustrate the differences in RIFR across varying pathological grades (A), IDH mutation status (B), EGFR expression levels (C), and 1p/19q codeletion (D). (E, F) Violin plots illustrate the differences in VFR across varying preoperative KPS scores (E) and the presence or absence of epilepsy (F). (G) Comparison of cortical thickness between ipsilesional and contralesional hemispheres in low‐ and high‐RIFR groups. (H, I) Comparison of FA and MD across low‐RIFR, high‐RIFR, and healthy controls (HCs) at the whole‐brain level and in tumor‐involved white matter tracts (CST, AF, SLF). (J–M) Spearman correlation scatter plots explore the relationships between the RIFR and brain network properties, including characteristic path length (J), clustering coefficient (K), local efficiency (L), and global efficiency (M). (N, O) Violin plots assess the differences in RIFR among patients with varying degrees of cognitive impairment (N, *n* = 79) and the presence or absence of language deficits (O, *n* = 85). Statistical significance is indicated by asterisks: ^*^ *p* < 0.05, ^**^ *p* < 0.01, ^***^ *p* < 0.001, illustrating the levels of correlation and difference across the explored variables. IDH, isocitrate dehydrogenase; EGFR, epidermal growth factor receptor; KPS, Karnofsky Performance Status; FA, fractional anisotropy; MD, mean diffusivity; HC, healthy control; CST, corticospinal tract; AF, arcuate fasciculus; SLF, superior longitudinal fasciculus.

In terms of clinical symptoms, no significant differences in RIFR were found between patients with a Karnofsky Performance Status (KPS) score below 90 and those 90 and above, or between patients with and without epilepsy (Figure S5). However, patients with worse clinical symptoms, such as lower KPS score and epilepsy, had a larger absolute volume of intratumoral functional regions (*p* < 0.01, Figure 5E,F). This indicates that as the absolute volume of intratumoral functional regions increases, it is more likely to lead to significant clinical manifestations. In addition, RIFR showed no strong correlation with patient age (*R* = −0.055, *p* = 0.336) or tumor volume (*R* = −0.127, *p* = 0.026) (Figure S9A; Figure S9B). Gliomas with higher and lower RIFR also did not differ significantly in their distribution across the whole brain (Figure S9C,D).

For peritumoral network analysis, similar conclusions were drawn across different thresholds (Figures S7 and S8). Tumor grade and IDH mutation status, as well as EGFR positivity, were found to be correlated with RPTR (Figure S7), whereas VTR was only affected by IDH status (Figure S8).

### 2.4 Structural and Diffusion Correlates of Functional Network Integration

To investigate whether functional network features reflect whole‐brain structural alterations, we analyzed cortical thickness and white matter microstructure. In the RIFR‐based stratification, no significant difference in cortical thickness between ipsilesional and contralesional hemispheres (*p* = 0.161) was observed in the low‐RIFR group. In contrast, the high‐RIFR group exhibited significantly increased cortical thickness in the ipsilesional hemisphere compared with the contralesional side (*p* < 0.001, Figure 5G). Diffusion MRI analysis revealed that both high and low RIFR groups showed reduced fractional anisotropy (FA) and increased mean diffusivity (MD) compared with healthy controls across the whole brain and major white matter tracts, consistent with widespread microstructural disruption in glioma patients (Figure 5H,I). However, no significant difference in FA was observed between high and low RIFR groups (Figure 5H). In contrast, MD values were significantly higher in the low‐RIFR group than in the high‐RIFR group across the whole brain and in the corticospinal tract (CST), arcuate fasciculus (AF), and superior longitudinal fasciculus (SLF) (Figure 5I), indicating more severe microstructural damage. Similar patterns were observed in RPTR‐based analyses (Figure S10). These findings suggest that lower RIFR and RPTR reflect structural disconnection and more extensive tissue damage.

### 2.5 Correlations of Network Features With Whole Brain Network Disruption and Brain Function

To further investigate whether intratumoral functional regions reflect glioma‐induced alterations in global brain functional network and whether these disruptions may contribute to higher‐order functions such as language and cognition, we first analyzed the correlations between RIFR and global brain network topology. As shown in Figure 5J–M, RIFR was positively correlated with characteristic path length (*R* = 0.328, *p* < 0.001), clustering coefficient (*R* = 0.490, *p* < 0.001), and local efficiency (*R* = 0.402, *p* < 0.001), while it was negatively correlated with global efficiency (*R* = −0.367, *p* < 0.001). These findings suggest that tumors with higher RIFR values—indicating more preserved intratumoral functional regions—are associated with network architectures that exhibit more segregated processing (e.g., higher clustering and local efficiency) and less integrated global communication (lower global efficiency).

Given the close association between functional networks and brain function, we further compared the differences in RIFR among patients with varying degrees of cognitive and language impairments. Cognitive functions assessed by the Montreal Cognitive Assessment (MoCA) revealed that patients with “moderate to severe” cognitive impairment had significantly lower RIFR compared with those in the “mild” or “normal” groups (*p* < 0.001, Figure 5N). Similarly, language impairments identified through the Boston Naming Test and Fluency Test were also associated with lower RIFR compared with patients with normal language functions (*p* = 0.039, Figure 5O). Using an alternative language network atlas, we specifically calculated the RIFR associated with the language network. The findings remained consistent, showing that patients with language impairments had significantly lower language network RIFR (Figure S11, *p* = 0.030). These results suggest that both cognitive and language impairments are associated with reduced RIFR.

For the PSA area (RPTR), similar experiments were repeated, and the results paralleled those of RIFR. RPTR also showed varying degrees of correlation with characteristic path length, clustering coefficient, local efficiency, and global efficiency, though the absolute values of correlation coefficients were smaller than those for RIFR (Figure S12A–D). In terms of brain function assessment, the “Moderate to severe” group showed significantly lower RPTR compared with the other groups (Figure S12E, *p* < 0.01), but there were no differences in RPTR between patients with and without language impairments (Figure S12F). These findings underscore the complex and varied landscape of intratumoral and peritumoral brain networks in glioma patients, highlighting the intricate interplay between tumor biology and the surrounding brain architecture.

### 2.6 Validation of Main Findings Across Parcellation Schemes and FC Thresholds

Given that the choice of brain parcellation schemes, FC thresholds, and atlas selection for graph‐theoretical analyses may influence the results, we performed additional validation analyses. The main findings remained highly consistent across different parcellations and FC thresholds (see Supporting Information Results; Figures S13–S15).

### 2.7 Prognostic Value of Glioma‐Related Functional Network Features

To evaluate whether glioma‐induced changes in brain networks could serve as prognostic indicators, we conducted univariate and multivariate regression analyses, incorporating patient characteristics such as sex, age, 2021 World Health Organization (WHO) grade [42], tumor volume, and functional connectivity features (RIFR, VFR, RPTR, and VTR). The analysis aimed to predict the two‐year survival status of patients. In the univariate analysis, significant factors influencing survival included age, WHO grade, RIFR, and RPTR (Figure 6A). The multivariate analysis adjusted for potential confounders further substantiated these findings. Grade IV gliomas remained a strong predictor of poorer survival, with an adjusted odds ratio of 8.930 (95% CI: 2.322–34.341, *p* = 0.001). The association of higher RIFR with better survival was also maintained in the multivariate model, with an adjusted odds ratio of 0.232 (95% CI: 0.059–0.912, *p* = 0.036). Further survival curve analyses were conducted by stratifying patients based on the median RIFR and RPTR. Patients with higher RIFR (Figure 6B, *p* = 0.004) or RPTR (Figure 6C, *p* = 0.029) demonstrated significantly better survival than those with lower RIFR or RPTR.

**FIGURE 6:**
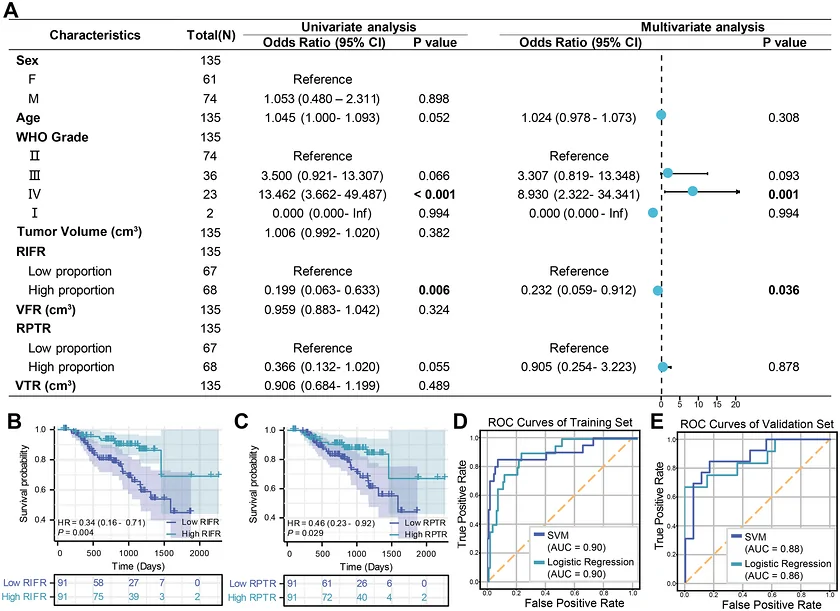
Prognostic value of glioma‐related functional network features. (A) A forest plot illustrating the results from univariate and multivariate analyses to identify factors impacting the 2‐year survival status of glioma patients. (B, C) Kaplan–Meier survival curves comparing the survival outcomes between patients with different RIFR and RPTR. (D, E) Features receiver operating characteristic (ROC) curves for prognostic models built using support vector machine (SVM) and logistic regression in the training and validation sets, based on features derived from glioma‐related brain functional network alterations. ROC, receiver operating characteristic; SVM, support vector machine.

### 2.8 Prognostic Model Prediction Based on Glioma‐Related Network Alterations

To assess the relationship between glioma‐related functional network features and patient prognosis, we employed machine learning models—support vector machine (SVM) and logistic regression—using variables from univariate regression analyses and the volumes of 17 brain network functional regions identified within tumors. First, we constructed baseline prediction models of two‐year survival status using only conventional clinical predictors (age, sex, tumor volume, and WHO grade). These models achieved AUCs of 0.79 (SVM) and 0.80 (logistic regression), respectively (Figure S16). When tumor‐related network features were added, model performance significantly improved. The SVM highlighted “WHO Grade”, “VFR”, “PSA area volume”, “Volume of N7”, and “Volume of N15” as key features, achieving an AUC of 0.90 (Figure 6D). The logistic regression model also achieved an AUC of 0.90 in the training dataset. In the external validation dataset, the SVM and logistic regression models achieved AUCs of 0.88 and 0.86 (Figure 6E), respectively, supporting their generalizability and predictive value.

### 2.9 Transcriptomic and Cellular Validation of Intratumoral Functional Heterogeneity

To further investigate the biological basis of imaging‐defined intratumoral functional regions, we performed integrative transcriptomic and cellular analyses. Using the Chinese Glioma Genome Atlas (CGGA) dataset, we identified 19 glioma patients with both fMRI and mRNA sequencing data. Based on RIFR, patients were stratified into high‐RIFR (*n* = 9) and low‐RIFR (*n* = 10) groups. Differential expression analysis followed by Gene Ontology (GO), Kyoto Encyclopedia of Genes and Genomes (KEGG), and Gene Set Enrichment Analysis (GSEA) revealed that high‐RIFR tumors were enriched in biological processes related to nervous system development, axonogenesis, and neurogenesis (Figure 7A). Molecular function analysis highlighted ion channel activity and transmembrane transport (Figure 7A). GSEA further demonstrated enrichment in synaptic signaling pathways, including neurexin–neuroligin interactions and Netrin‐1 signaling, and lipid metabolism pathways mediated by SREBP (Figure 7B). To validate these findings at the cellular level, we performed spatially guided sampling and single‐cell RNA sequencing in four glioma patients. Intratumoral functional regions exhibited significantly increased proportions of macrophages (30.25 ± 9.77 vs. 4.40 ± 1.65, *p* = 0.012) and T cells (12.60 ± 6.78 vs. 1.65 ± 0.89, *p* = 0.037) compared with nonfunctional regions (Figure 7C,D). Together, these results suggest that intratumoral functional regions represent biologically distinct tumor subregions characterized by enhanced neuronal interaction, metabolic activity, and immune involvement.

**FIGURE 7:**
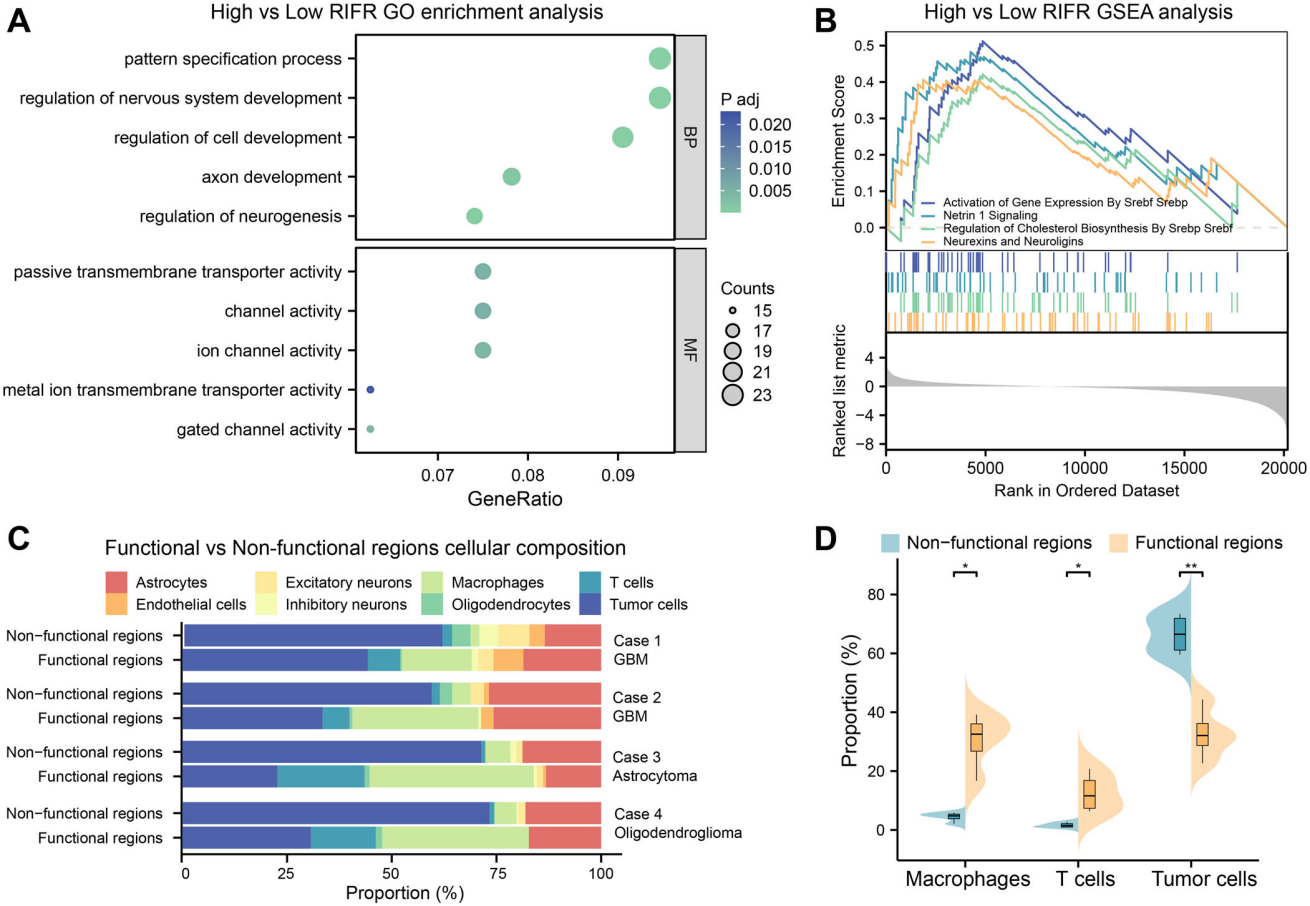
Imaging—molecular and cellular validation of intratumoral functional regions. (A, B) GO enrichment and GSEA analyses comparing high‐RIFR and low‐RIFR tumors using CGGA mRNA sequencing data. (C, D) Comparison of cellular composition between functional and nonfunctional tumor regions based on single‐cell RNA sequencing. GO, Gene Ontology; GSEA, gene set enrichment analysis; CGGA, Chinese Glioma Genome Atlas; mRNA, messenger RNA.

## 3 Discussion

This study provides a comprehensive analysis of glioma‐induced brain network alterations by integrating individualized functional connectivity analysis with detailed clinical and molecular profiling. By examining both intratumoral and peritumoral functional networks, we identified patterns of functional connectivity and their associations with tumor characteristics, clinical symptoms, and patient outcomes.

Advances in neuro‐oncology increasingly emphasize the need for personalized approaches in the management and treatment of gliomas, due to their profound impact on brain networks. Initial studies in glioma patients utilized healthy controls to highlight differences in brain connectivity [17], employing indices like “Abnormality Index” to capture disruptions across whole brain networks in glioma patients [12]. Such methods, while useful, often oversimplify the complex interactions and network changes caused by gliomas. Subsequent research evolved to more sophisticated models, with studies like those by Daniel et al. [18], which introduced the concept of mapping intratumoral functional areas. However, these did not account for tumor‐induced brain reorganization, potentially skewing their accuracy. Our previous approach builds upon these methods, integrating an iterative individual parcellation technique that enhances precision but has been traditionally limited by computational intensity and applicability to tumors affecting both brain hemispheres [11]. The methodology we have developed and validated through this study addresses these limitations by efficiently delineating tumor‐impacted and peritumoral brain networks at a voxel‐level resolution. By setting various FC thresholds (Figures S5–S8), our results not only demonstrated consistency but also corroborated DCS findings (Figure 4), enhancing their reliability. Furthermore, the observed correlation of intratumoral functional regions with clinical and cognitive outcomes underscores the potential of this approach in clinical settings.

Additionally, our method offers good biological interpretability. Brain dysconnectivity describes how localized damage or anomalies can result in widespread network disruptions, leading to a loss of synchrony between different brain regions [19, 20]. In our study, focusing on the connectivity of tumor‐impacted areas with intact brain networks has illuminated the dual nature of glioma as both a disruptor and an unexpected integrator within the brain's interconnected landscape. Our results suggest that gliomas with more preserved intratumoral and peritumoral functional regions (higher RIFR and RPTR) tend to maintain local network integrity, as reflected by increased clustering and local efficiency (Figure 5J–M). However, they may simultaneously disrupt global network communication, leading to increased characteristic path length and reduced global efficiency. This duality reflects a localized compensatory remodeling in response to tumor‐induced damage, at the cost of reduced global integration—consistent with the theory of functional segregation and integration imbalance in network neuroscience. These findings highlight the dual role of gliomas in both disrupting and integrating with brain networks, suggesting that intratumoral functional regions may serve as indicators of tumor‐induced network remodeling.

Previous studies have reported the presence of functional regions within glioblastoma (GBM) [12], but our findings expand upon these initial observations. Our results also include Grade I–III gliomas, showing that the proportion of intratumoral functional regions is often higher in Grade I–III gliomas compared with GBMs (Figure 5A). Additionally, in molecular subtypes, we found that tumors with IDH wild‐type status or EGFR amplification exhibited reduced RIFR (Figure 5B,C). Overall, the higher the malignancy grade of the tumor, the fewer functional areas are retained within the tumor. This difference can be attributed to several factors. First, highly malignant tumors, such as GBMs, are characterized by a larger extent of necrotic tissue, while low‐grade gliomas generally contain a higher density of viable tumor cells centrally, which gradually decreases toward the tumor periphery [21, 22]. Secondly, glioma cells from different molecular subtypes may exhibit distinct patterns of interaction with neural circuits [6, 23, 24]. Supporting evidence for this is found in the fact that IDH wild‐type gliomas have a more significant impact on the brain network compared with IDH‐mutant gliomas, even affecting widespread functional networks in the contralesional hemisphere [12]. Other studies have also shown that reorganization of the default mode network (DMN) is more pronounced in gliomas with IDH mutations, suggesting that this subtype is associated with greater stability in functional connectivity and, consequently, better cognitive performance [25, 26]. Our findings highlight the importance of distinguishing between glioma subtypes when studying brain network changes in future research.

Moreover, we observed that the local network features of gliomas were not associated with tumor volume, location, or patient age, but were closely related to clinical manifestations, including KPS scores, the presence of epilepsy, changes in whole‐brain structure and network characteristics, cognitive and language function, and overall survival (Figures 5 and 6). Viewing brain network alterations as a bridge helps to explain how tumor pathology and molecular characteristics influence brain networks, thereby leading to various patient symptoms and outcomes [7, 27]. It is now well established that neuron–tumor networks within gliomas can promote tumorigenesis and influence both radiosensitivity and patient prognosis [28, 29]. Building on this understanding, targeting glioma–neuron neurotransmitter communication has emerged as a promising therapeutic strategy [15]. These insights underscore the importance of integrating molecular profiling with network neuroscience to enhance the understanding of glioma pathophysiology and optimize patient‐specific management strategies [30].

Another significant finding of our study is the role of brain network changes as important predictors of patient outcomes in glioma. Prediction models based on connectomics have been widely applied in neuropsychiatric diseases [31], such as predicting atrophy progression in Parkinson's disease [32], accelerated functional brain aging in preclinical Alzheimer's disease [33], and distinguishing bipolar disorder from major depressive disorder in adolescents [34]. However, such studies are still in their early stages in the context of gliomas. Previous research has largely focused on identifying correlations between functional connectivity and patient‐specific symptoms, such as language impairment [35], types of epilepsy [36], recovery status [37], or general prognosis. In our study, we have taken a step forward by using a larger cohort to construct machine learning models that predict patient survival based on brain network properties, achieving high accuracy in both training and validation sets. This approach demonstrates that network‐level disruptions caused by gliomas have tangible prognostic implications that can be quantitatively assessed.

In addition to functional connectivity, our prior research has shown that structural changes and remodeling of the brain also have predictive value. For example, in patients with gliomas involving functional areas, the compensatory volumetric changes in the supplementary motor area correlated with postoperative motor function recovery [38]. This finding suggests that integrating structural and functional connectome features could further enhance our ability to assess and predict patient recovery and prognosis. This approach holds immense potential for clinical application, from guiding personalized rehabilitation strategies to predicting overall prognosis, ultimately improving patient outcomes through tailored interventions based on individual network characteristics.

Our integrative analyses provide mechanistic insight into the biological basis of intratumoral functional regions. High‐RIFR gliomas exhibit enrichment in gene programs related to neurodevelopment, synaptic organization, and ion channel activity, suggesting a “neuroactive” tumor phenotype. Specifically, activation of SREBP‐mediated cholesterol biosynthesis may provide essential components for membrane formation and synaptogenesis, while axon guidance molecules such as Netrin‐1 and synaptic adhesion molecules including Neurexins and Neuroligins facilitate the formation of tumor–neuron synaptic‐like connections. These connections enable neuronal electrical activity to be transmitted into tumor networks through ion channel‐mediated mechanisms, thereby promoting tumor proliferation and invasion.

Furthermore, the increased presence of macrophages and T cells in intratumoral functional regions suggests that immune cells may actively participate in this neuron–tumor interaction. Recent studies have demonstrated that glioma–neuronal circuit remodeling is closely linked to regional immunosuppression, characterized by altered macrophage polarization and T‐cell infiltration [39], and that neuron–immune–tumor crosstalk plays a critical role in glioma progression [40]. Together, these findings support a model in which intratumoral functional regions represent highly interactive niches integrating neuronal activity, metabolic support, and immune modulation, ultimately driving tumor progression.

One potential consequence of glioma–neuron interactions is large‐scale network remodeling. In this context, RIFR and RPTR may reflect the degree of tumor integration into whole‐brain networks, with higher values indicating greater coupling and lower values suggesting network isolation. Consistently, patients with higher RIFR/RPTR showed increased cortical thickness in the ipsilesional hemisphere (Figure 5G), which may reflect adaptive neuroplasticity driven by sustained tumor–brain interactions. Previous studies have reported similar findings, particularly in slower‐growing gliomas, where prolonged disease course may promote cortical thickening through enhanced plasticity [41, 42].

Despite the promising findings, our study has certain limitations that should be acknowledged. First, RIFR and RPTR are derived from blood oxygenation level‐dependent (BOLD)‐based functional connectivity, which reflects hemodynamic responses rather than direct neuronal activity [44]. In tumor tissue, neurovascular uncoupling, abnormal angiogenesis, and metabolic alterations may distort BOLD signals. Therefore, the observed functional connectivity patterns may be influenced by both neuronal and vascular factors and should be interpreted at the level of association rather than direct neural interactions. Although the spatial correspondence between intratumoral functional regions and canonical brain networks suggests partially preserved neurovascular coupling, we cannot exclude vascular or metabolic confounds. Second, the absence of perfusion‐weighted imaging (PWI) and susceptibility‐weighted imaging (SWI) limits our ability to further characterize vascular and hemodynamic contributions. Increased perfusion, susceptibility effects, and tumor‐related heterogeneity—including necrosis—may influence FC signals, particularly in high‐grade gliomas [44]. Future work should incorporate multimodal approaches, including breath‐hold or calibrated fMRI to assess neurovascular coupling, perfusion imaging to evaluate vascular contributions, and electrophysiological techniques such as electroencephalography (EEG) or magnetoencephalography (MEG) to better distinguish neuronal from vascular signals. Third, tumor mass effect may affect atlas‐based analyses. Although lesion‐masked spatial normalization was applied to mitigate this issue, residual distortion may still influence parcellation accuracy and connectivity estimation. Fourth, not all patients had complete molecular, clinical, and outcome data, which may introduce heterogeneity into the analysis. Fifth, the current DCS validation was limited to sensorimotor‐positive sites, restricting validation of other functional domains such as language. Finally, this study relied on preoperative fMRI data only. Longitudinal studies incorporating postoperative and follow‐up imaging would be valuable for assessing dynamic changes in brain networks.

In this study, we applied an individualized brain network mapping approach to characterize intra‐ and peritumoral functional reorganization using fMRI. Our findings demonstrate that gliomas induce distinct alterations in functional networks, which correlate with tumor pathology, clinical symptoms, structural alterations, whole‐brain network disruption, cognitive and language impairments, and survival outcomes. Network‐based features derived from our approach showed prognostic potential. These results deepen our understanding of gliomas as disorders of brain network architecture and highlight the promise of functional connectome analysis in guiding personalized diagnosis and treatment strategies.

## 4 Methods And Materials

### 4.1 Patients and Data Collection

This study aimed to characterize individualized intratumoral and peritumoral functional connectivity features and evaluate their correlation with molecular subtypes, brain structural alterations, network properties, and patient prognosis. A retrospective analysis was performed on all glioma patients who underwent rs‐fMRI before surgery at Beijing Tiantan Hospital (*n* = 309) and Harbin Medical University Cancer Hospital (*n* = 67) from June 1, 2016, to March 1, 2023. The inclusion criteria were as follows: (1) primary glioma confirmed by surgical pathology; (2) availability of preoperative T1‐weighted (T1w), T2‐weighted (T2w), fluid attenuation inversion recovery (FLAIR), and fMRI data; (3) Age ≥ 18 years. The presence of any one of the following exclusion criteria led to exclusion of the subject: (1) brainstem or cerebellar gliomas; (2) cystic glioma (no obvious solid content); (3) unclear tumor boundaries due to ambiguous enhancing margins on contrast‐enhanced T1w images or poorly defined T2w/FLAIR abnormalities; (4) poor image quality (low resolution, significant head motion, and metal artifacts); (5) history of psychiatric or neurological disorders. Of the 376 patients initially screened, 306 were ultimately included in the final analysis, and the screening process is outlined in Figure 1. For each patient, the following data were obtained: sex, age, preoperative status (presence of epilepsy, KPS score, and scores from cognitive and language function assessment scales), and prognostic information. Following the 2021 edition of the WHO Classification of Tumors of the CNS [45, 46], we gathered pathological information, including the WHO grade, as well as key molecular characteristics that are significant for classification and prognosis. These molecular characteristics included the presence of IDH‐1 mutation, 1p/19q co‐deletion, MGMT promoter methylation, EGFR amplification, TERT promoter mutation, ATRX mutation, and Ki‐67 expression levels. This study was conducted in accordance with the Declaration of Helsinki. The Ethics Committees of Beijing Tiantan Hospital and Harbin Medical University Cancer Hospital reviewed and approved a waiver of written informed consent.

### 4.2 MRI Data Processing

Preoperative MRI data, including T1w, T2w, FLAIR, and T1w postcontrast sequences, as well as fMRI data, were collected from two independent centers. Detailed acquisition parameters for these sequences are provided in the Supporting Information Materials. Tumor lesions were manually segmented into the core tumor, including both enhancing and nonenhancing portions (with necrosis), and the PSA area, which encompasses both edema and infiltrative tumor, particularly in high‐grade gliomas. This delineation was performed by two neuroradiologists (each with 6 years of experience), blinded to the patients’ clinical information. In cases where the discrepancy between their delineations exceeded 5% of the tumor mask pixel volume, a third radiologist with 20 years of experience in neuroradiology was consulted for confirmation. FMRIPrep was used to preprocess rs‐fMRI data involving anatomical and functional normalization, nuisance regression, band‐pass filtering, spatial smoothing, and quality assurance [47]. Detailed methods are provided in the Supporting Information Materials. To mitigate the technical variability arising from the use of multiple MRI scanners across different centers, the ComBat method was applied to imaging‐derived features, adjusting for site effects while preserving biological covariates such as age and sex for harmonization [48]. By modeling the scanner‐specific effects and normalizing across different batches, ComBat is effective at reducing systematic differences and enhancing comparability.

### 4.3 Individualized Intratumoral and Peritumoral Functional Connectivity Analysis

Early methods for calculating FC within gliomas did not account for the potential functional and structural remodeling of peritumoral tissue caused by the tumor [18]. To address this issue, we previously proposed an iterative individual parcellation approach [11]. However, this method was complex and difficult to generalize, requiring tumors to be confined to one cerebral hemisphere and was not widely used. In this study, we propose a simplified method for individualized functional connectivity analysis in glioma patients. This method addresses the shortcomings of previous approaches while simultaneously mapping changes in both intratumoral and peritumoral functional networks. The specific analytical methods of intra‐ and peritumoral networks are as follows (Figure 2):

**(1) Tumor subregion segmentation**: Two board‐certified neuroradiologists segmented the tumor into the core tumor portion and the PSA area using MRIcron software. Brain regions outside these areas, considered minimally affected by the tumor, were treated as normal‐appearing brain (NAB) regions.

**(2) Mapping functional networks in normal brain regions**: We used the Yeo 17 RSNs atlas [49], a widely used template previously derived from a group‐level analysis of 1000 subjects, as the basis for brain network parcellation. To minimize the impact of tumor‐induced mass effect, we employed lesion‐masked registration. The core tumor and PSA areas were excluded from the cost function during anatomical‐to‐standard space registration. Only the remaining NAB was nonlinearly registered to the Montreal Neurological Institute (MNI) 152 template, and the Yeo 17 network atlas was also transformed to this standard space to ensure anatomical correspondence.

**(3) Tumor network**: The core tumor portion was treated as an independent network, resulting in a total of 18 networks (17 normal RSNs + 1 tumor network). After this parcellation, voxels within the tumor may exhibit strong correlations with normal RSNs, and voxels in the PSA area may show strong correlations with the tumor network.

**(4) Functional connectivity analysis**: FC values were calculated using the Spearman correlation coefficient between voxels and brain networks. A threshold was used to remove FC values (detailed below). Voxels where the FC with any of the 17 canonical RSNs or the tumor network exceeded the threshold were operationally defined as “functionally connected”. When a tumor voxel demonstrated suprathreshold connectivity with multiple RSNs, it was assigned to the network with the highest FC value

**(5) Functional mapping of intratumoral networks**: We identified intratumoral functional regions by calculating voxel‐wise Spearman correlation between the time series of each tumor voxel and the average time series of each of the 17 canonical RSNs. Voxels with a correlation coefficient greater than the threshold with any RSN were considered functionally connected and labeled as intratumoral functional regions. The VFR in core tumors and the ratio of the intratumoral functional regions (RIFR) to core tumor volume were calculated:$\;\mathrm{RIFR}=\frac{\mathrm{The}\;\mathrm{volume}\;\mathrm{of}\;\mathrm{the}\;\mathrm{functional}\;\mathrm{regions}\;\mathrm{in}\;\mathrm{core}\;\mathrm{tumor}\;(\mathrm{VFR})}{\mathrm{Core}\;\mathrm{tumor}\;\mathrm{volume}}\;$.

**(6) Functional mapping of peritumoral networks**: The PSA region may contain voxels with functional connections to either the core tumor network or normal brain networks. In this analysis, we specifically focused on PSA voxels that exhibited functional connectivity to the core tumor network. The volume of the tumoral regions (VTR) functionally related to the tumor network in the PSA area and the ratio of the peritumoral tumoral regions (RPTR) to PSA volume were calculated: $\mathrm{RPTR}=\frac{\mathrm{The}\;\mathrm{volume}\;\mathrm{of}\;\mathrm{the}\;\mathrm{tumoral}\;\mathrm{regions}\;\mathrm{in}\;\mathrm{PSA}\;\mathrm{area}\;(\mathrm{VTR})}{\mathrm{PSA}\;\mathrm{volume}}\;$.

The choice of functional connectivity (FC) threshold can influence the identification of intratumoral functional regions. To assess robustness, we evaluated thresholds ranging from 0.3 to 0.7 at 0.1 intervals. As the threshold increased, VFR, RIFR, VTR, and RPTR decreased, reflecting progressively stricter voxel selection (Figure S17). Higher thresholds preferentially retain stronger and more reliable connections while reducing potential noise. Therefore, to balance specificity and robustness, we selected an FC threshold of 0.7, consistent with the convention that Spearman correlation coefficients above 0.7 indicate strong associations. Results of repeated validation under other thresholds are provided in Figures S5–S8, S13, and S14.

### 4.4 DCS Mapping During Awake Surgery

In our study, seven patients had tumors located in the sensorimotor cortex. To accurately delineate functional boundaries during surgery, DCS was employed under awake conditions. DCS was performed using an Ojemann Cortical Stimulator (Integra Life, Plainsboro, NJ, USA) that delivered 60 Hz biphasic pulse trains. The stimulation parameters included a square wave with a pulse duration of 1 s. Starting at an intensity of 1 mA, the stimulation was incrementally increased by 0.5 mA steps. The procedure was carefully monitored, and the stimulation was ceased either upon the induction of involuntary motor movements or sensory disturbances (Figure S4), adhering to the 2/3 principle [50], or when the intensity reached a maximum of 6 mA. Locations eliciting positive responses were marked with 5 mm fiducial markers and subsequently mapped into the MNI space for further analysis.

### 4.5 Analysis of Clinical and Pathological Factors

We analyzed the relationship between the extracted quantitative metrics of the glioma‐related network (VFR, RIFR, VTR, and RPTR) and clinical and pathological factors. The Shapiro–Wilk normality test was employed to assess the normality of data distribution. When categorizing patients into two groups based on tumor grade, molecular characteristics, and presence or absence of epilepsy, we performed Levene's test for homogeneity of variances followed by the Wilcoxon rank‐sum test. For continuous variables such as age and tumor volume, we utilized the Spearman correlation method to analyze their relationship with the metrics of interest.

### 4.6 Cortical Thickness Analysis

Patients with tumors confined to a single hemisphere (*n* = 240) were included in the cortical thickness analysis to avoid confounding effects of bilateral lesions. Subjects were stratified into high‐ and low‐RIFR/RPTR groups based on median values. High‐resolution T1‐weighted images were processed using FreeSurfer, following standard procedures including skull stripping, intensity normalization, and cortical surface reconstruction [51]. Cortical thickness was computed as the distance between the gray matter–white matter boundary and the pial surface. Regional cortical thickness values were extracted based on the Desikan–Killiany atlas for both hemispheres. For each subject, mean cortical thickness of the ipsilesional and contralesional hemispheres was calculated. Paired *t*‐tests were used to compare hemispheric differences within each group.

### 4.7 Diffusion MRI Analysis

A total of 280 glioma patients in our cohort had preoperative diffusion tensor imaging (DTI) data available and were included in the diffusion analysis. In addition, 48 age‐ and sex‐matched healthy controls (HCs) were included for comparison. Diffusion MRI data were preprocessed using standard pipelines, including correction for eddy currents and head motion, followed by tensor fitting to derive diffusion metrics. FA and MD maps were calculated for each subject. White matter tractography was performed using the XTRACT toolbox [52], which provides standardized protocols for automated reconstruction of major white matter pathways. Whole‐brain FA and MD values were obtained. Tract‐specific analyses were performed for functionally relevant tracts, including the corticospinal tract (CST), arcuate fasciculus (AF), and superior longitudinal fasciculus (SLF). A tract was considered tumor‐involved when the tumor mask overlapped with the corresponding tract in a standard white matter atlas. For these tracts, FA and MD values were further analyzed. Group comparisons were performed across high‐ and low‐RIFR/RPTR groups and healthy controls. Differences in FA and MD were evaluated at both whole‐brain and tract‐specific levels using appropriate statistical tests, with significance set at *p* < 0.05.

### 4.8 Analysis of Network Properties

To investigate whether changes in intratumor and peritumor brain networks are associated with alterations in network properties, we selected graph theory metrics that could reflect the disruption of the brain connectome, including characteristic path length, clustering coefficient, local efficiency, and global efficiency, as observation indices. The specific meanings of the brain network metrics are detailed in the Supporting Information Materials. We used the Human Brainnetome Atlas (210 cortical and 36 subcortical subregions, http://atlas.brainnetome.org) for brain parcellation and calculated the average values of these metrics across the whole brain for each patient [49, 53], performing Spearman correlation analysis with RIFR and RPTR. Given that multiple network measures were tested, false discovery rate (FDR) correction was applied to control for multiple comparisons. Adjusted *p*‐values < 0.05 were considered statistically significant. The Yeo 17 atlas was not used for this analysis because its large‐scale parcellations may not fully capture regional brain network alterations.

### 4.9 Cognitive and Language Function Assessment

Preoperative cognitive function (*n* = 79) and language function (*n* = 85) assessments were conducted for some patients. The MoCA is a widely used tool for detecting cognitive impairments, covering domains such as attention, concentration, memory, language, visuospatial skills, conceptual thinking, calculation, and orientation [54]. Patients were scored preoperatively using the MoCA, with a total possible score of 30 points. For patients with less than 12 years of education, an additional point was added to their original MoCA score to correct for the impact of educational level on cognitive test outcomes [55]. Based on their scores, patients were categorized into three groups: normal cognitive function (26 points and above), mild cognitive impairment (18–25 points), and moderate to severe cognitive impairment (17 points and below).

Language assessment included the Fluency Test and the Boston Naming Test [56]. In the Fluency Test, patients were required to name as many words as possible beginning with a common Chinese character within one minute; naming 11 or more words was considered normal. In the Boston Naming Test, correctly naming 25 or more out of the given 30 pictures was considered normal. Patients with any abnormality in either language function test were classified as having impaired language function, while those who performed normally on both tests were classified as having normal language function. Since the Yeo 17 atlas does not include an independent language network, we incorporated a language network atlas derived from fMRI data of over 800 healthy individuals [57]. We then calculated the RIFR of tumor regions functionally connected to the language network across different FC thresholds.

### 4.10 Survival Analysis

A total of 182 patients with available survival data were included in the survival analysis. Factors considered in the univariate and multivariate regression analyses included sex, age, WHO grade, tumor volume, RIFR, VFR, RPTR, and VTR. The median survival time of the cohort was approximately 2 years, and therefore, the 2‐year survival status served as the primary endpoint for observation. The statistical analysis was conducted using R software (version 4.2.1) with packages “rms” (version 6.4.0) and “ResourceSelection” (version 0.3‐5). Variables with a *p*‐value less than 0.1 in the univariate analysis were included in the multivariate binary logistic regression to visualize potential associations with the survival outcome. Subsequently, patients were grouped based on the median values of RIFR and RPTR for survival curve analysis. The survival curves were plotted and analyzed using the “survival” package, testing the proportionality of hazards assumption and fitting the survival regression model using Cox regression.

### 4.11 Prognostic Model Development

To evaluate the prognostic value of brain network changes in glioma patients, we utilized two machine learning models, SVM and Logistic Regression, to analyze survival status at 2 years. Key indices such as demographic details, clinical status, WHO grade, and brain network‐related properties were considered. The SVM utilized recursive feature elimination with cross‐validation (RFECV) was performed by the “e1071” package in R software (version 4.2.1), to optimize feature selection [58], targeting a minimal set of 5 features. Data normalization was executed before model training to enhance model performance. For the logistic regression model, hyperparameter optimization was performed by GridSearchCV in Python (version 3.8). The model was tuned by evaluating different values of regularization strength, solvers, and maximum iterations. In addition, we collected follow‐up data of 31 glioma patients from the Beijing Neurosurgical Institute as an independent validation cohort in our model predicting 2‐year survival status. These patients were age‐ and sex‐matched to our primary cohort and underwent fMRI examinations at the Beijing Neurosurgical Institute. Their inclusion and exclusion criteria were identical to those of the primary dataset, and they subsequently received treatment either at Tiantan Hospital or other medical centers. Receiver operating characteristic (ROC) curves were used to assess the diagnostic performance of two models in the training and validation sets.

### 4.12 Imaging–Transcriptomic Characterization of Intratumoral Functional Heterogeneity

To investigate the molecular characteristics associated with intratumoral functional regions, we performed an imaging–transcriptomic analysis using data from the CGGA database (https://www.cgga.org.cn/) [59]. Patients with both fMRI data and mRNA sequencing data were identified (*n* = 19). fMRI data were processed using the same pipeline as described for the primary cohort. RIFR was calculated for each patient, and subjects were stratified into high‐RIFR (*n* = 9) and low‐RIFR (*n* = 10) groups based on the median split. Differential gene expression analysis between the two groups was conducted using the clusterProfiler package in R software. GSEA was performed using ranked gene lists based on log fold change values, with significance thresholds set at adjusted *p* < 0.05 and *q*‐value < 0.25. In addition, genes with |logFC| > 1 and adjusted *p* < 0.05 were subjected to GO and KEGG pathway enrichment analyses. Only pathways with adjusted *p* < 0.05 and gene counts greater than 15 were retained.

### 4.13 Spatially Guided Sampling and Single‐Cell Transcriptomic Profiling

To further investigate the biological basis of intratumoral functional regions, we performed spatially guided tissue sampling combined with single‐cell transcriptomic profiling. Four glioma patients in our dataset were derived from a prospective study designed to investigate longitudinal brain‐network changes before and after treatment. The study was registered in the National Medical Research Registration and Filing Information System (registration no. MR‐11‐23‐033670). The present analysis used the preoperative imaging and intraoperative tissue‐sampling component of this prospective study. All four patients underwent preoperative resting‐state fMRI, from which intratumoral functional and nonfunctional regions were identified using the functional connectivity‐based framework described above. During surgery, neuronavigation was employed to accurately localize these regions, enabling region‐specific tissue sampling. All sampling was performed within the clinically indicated surgical field and within the planned extent of tumor resection. The imaging‐guided, neuronavigation‐assisted sampling did not alter the planned surgical procedure or standard clinical management and did not introduce additional surgical risk. No tissue was resected solely for research purposes; all specimens used for research were obtained from tissue that would otherwise have been removed as part of the clinically indicated tumor resection. Fresh tumor specimens were dissociated into single‐cell suspensions using enzymatic digestion and mechanical dissociation. Following quality control and removal of low‐quality cells, single‐cell RNA sequencing libraries were generated and sequenced according to standard protocols [60]. Cell populations were identified based on canonical marker genes and classified into major cell types, including tumor cells, astrocytes, oligodendrocytes, endothelial cells, excitatory neurons, inhibitory neurons, macrophages, and T cells. Relative proportions of these cell populations were compared between intratumoral functional and nonfunctional regions.

### 4.14 Statistical Analyses

Data processing and statistical analyses were performed using R software version 4.2.1. The most important R packages used herein include ggplot2, version 3.3.6, for data visualization; and stats, version 4.2.1, and car, version 3.1‐0, for the corresponding statistical testing. The statistical analysis process includes a direct comparison between two groups, with the *t*‐test applied if the data are normally distributed with equal variances. In a situation where the data are normally distributed but their variances are not, Welch's *t*‐test is applied. Nonparametric tests include the Wilcoxon rank‐sum test when there is no normal distribution of data. For the comparison among multiple groups, our data were found to be normally distributed but failed to meet the assumption of homogeneity of variance; hence, we conducted Welch's one‐way ANOVA. Wilcoxon signed‐rank test is used for paired data when differences do not follow a normal distribution. Spearman's correlation analysis is used to explore relationships between variables. The absolute value of the correlation coefficient is used to interpret the strength of the relationship. All statistical hypothesis tests are conducted at a significance level of 0.05.

## Author Contributions

**Hongbo Bao**: Conceptualization, formal analysis, funding acquisition, methodology, validation, visualization, Writing – original draft preparation. **Yan Bai**: Formal analysis, methodology, writing – original draft preparation. **Jie Hu**: Formal analysis, methodology. **Wencai Ding**: Formal analysis, methodology, resources. **Jiacheng Lai**: Investigation, resources. **Andrew Zalesky**: Conceptualization, supervision, Writing – review and editing. **Ye Ella Tian**: Supervision, writing – review and editing. **Yanmei Tie**: Resources; Writing – review and editing. **Janet Mei**: Supervision, Writing – review and editing. **Shengyu Fang**: Funding acquisition, resources. **Xing Fan**: Investigation, resources. **Licia P. Luna**: Supervision, writing – review and editing. **Craig K. Jones**: Supervision; Writing – review and editing. **Jianxun Ren**: Supervision, Writing – review and editing. **Weigang Cui**: Supervision, writing – review and editing. **Jiadong Yan**: Methodology, writing – review and editing. **Zeya Yan**: Investigation, resources. **Zihan Wang**: Investigation, resources. **Renwu Zhang**: Investigation, resources. **Zhonghua Lv**: Funding acquisition. **Yihang Jiao**: Methodology, resources, Writing – review and editing. **Tao Jiang**: Conceptualization, supervision, writing – review and editing. **Peng Liang**: Conceptualization, funding acquisition, supervision, writing – review and editing. **Haris I. Sair**: Conceptualization, supervision, writing – review and editing. **Yinyan Wang**: Conceptualization, funding acquisition, supervision, Writing – review and editing. All authors have read and approved the final manuscript.

## Ethics Statement

This study was conducted in accordance with the Declaration of Helsinki and approved by the Ethics Committees of Beijing Tiantan Hospital and Harbin Medical University Cancer Hospital (JS2024‐58, KY2025‐269‐03, KY2026‐229‐03). As this study was a retrospective analysis, the Ethics Committees of Beijing Tiantan Hospital and Harbin Medical University Cancer Hospital reviewed and approved a waiver of written informed consent. For patients who had previously provided written informed consent, consent was obtained and documented. For patients whose written informed consent was waived, oral notification was provided during follow‐up in accordance with the Ethics Committee's requirements. The tissue samples used in this study included a subset derived from a prospective study registered in the National Medical Research Registration and Filing Information System (registration no. MR‐11‐23‐033670).

## Conflicts of Interest

The authors declare no conflicts of interest.

## Supporting information




**Supporting File**: mco271083‐sup‐0001‐SuppMat.docx

## Data Availability

Clinical data, imaging data, and biological samples used in this study were de‐identified or coded before analysis. Direct personal identifiers were removed and replaced with unique study identifiers to protect participant privacy and confidentiality. Data related to direct cortical stimulation are publicly available through the China Functional Brain Atlas (https://cfbm.cibr.ac.cn/#/home). RNA‐sequencing data and a subset of the associated imaging, pathological, and follow‐up data are publicly available through the Chinese Glioma Genome Atlas (https://www.cgga.org.cn/). These publicly available datasets can be accessed in accordance with the respective database policies. Other de‐identified participant‐level clinical and imaging data supporting the findings of this study may be available from the corresponding author upon reasonable request, subject to applicable ethics approval, institutional data‐governance requirements, and data‐use agreements. The remaining individual‐level data and biological materials are not publicly available because unrestricted public disclosure is not permitted under participant consent, privacy protection requirements, and institutional and ethical regulations governing these data and materials.
