# Supplementary material for: Intratumoral and Peritumoral Functional Connectivity Reveals Network‐Level Signatures of Glioma
Source: MedComm (2020). 2026 Oct 17;7(11):e71083. doi: 10.1002/mco2.71083 (PMC13675729; doi:10.1002/mco2.71083)
Supplement: Supplementary file 1 [file MCO2-7-e71083-s001.docx]

Supplementary Materials for

**Intratumoral and Peritumoral Functional Integrations Reveals Network-Level Signatures of Glioma**

Hongbo Bao *et al.*

*Corresponding authors:

Yinyan Wang, E-mail: [tiantanyinyan@126.com](mailto:tiantanyinyan@126.com);

Haris I. Sair, E-mail: [hsair1@jhmi.edu](mailto:hsair1@jhmi.edu);

Peng Liang, E-mail: [liangpenghmu@hotmail.com](mailto:liangpenghmu@hotmail.com).

**1. Supplementary Materials and Methods**

**1.1. MRI Acquisition Parameters**

In Beijing Tiantan Hospital and Beijing Neurosurgical Institute, MRI data were acquired using a Prisma 3.0-T MR scanner (Siemens, Erlangen, Germany). Anatomical T1-weighted images were obtained using a magnetization-prepared rapid acquisition gradient echo (MPRAGE) sequence with the following parameters: repetition time (TR) = 2300 ms, echo time (TE) = 2.3 ms, flip angle (FA) = 8°, field of view (FOV) = 220 × 220 mm, voxel size = 1.0 × 1.0 × 1.0 mm, and number of slices = 192. T2-FLAIR images were captured using a fluid-attenuated inversion recovery sequence with TR = 3200 ms, TE = 87 ms, FA = 150°, FOV = 220 × 220 mm, voxel size = 0.9 × 0.9 × 5.0 mm, and number of slices = 25. rs-fMRI parameters included TR = 2000 ms, TE = 30 ms, FA = 75°, FOV = 220 × 220 mm, voxel size = 3.0 × 3.0 × 5.0 mm, number of slices = 30, and acquisition duration of 8 minutes. Diffusion magnetic resonance imaging data were acquired using a single-shot echo-planar imaging sequence with TR = 8000 ms, TE = 60 ms, FOV = 282 × 282 mm, voxel size = 2.2 × 2.2 × 2.0 mm, directions = 30, b-values = 0/1000 s/mm², and slices = 75.

In Harbin Medical University Cancer Hospital, MRI data were acquired using a SIGNA 3.0-T scanner (GE, USA). T1-weighted images were captured with TE = 3.2 ms, TR = 6.9 ms, FOV = 240 × 240 mm, voxel size = 1.2 × 1.2 × 1.2 mm, and number of slices = 160 slices. FLAIR imaging parameters included TE = 100 ms, TR = 8000 ms, FOV = 250 × 250 mm, voxel size = 1 × 1 × 1 mm, and number of slices = 360 slices. fMRI was performed with TE = 35 ms, TR = 1600 ms, FOV = 230 × 230 mm, voxel size = 2 × 2 × 2 mm, and number of slices = 72 slices, and acquisition duration of 12 minutes. In both centers, gadopentetate dimeglumine was used as the contrast agent at a dose of 0.1 mmol/kg body weight.

**1.2. fMRI Preprocessing**

The primary preprocessing steps included: (1) Anatomical processing: skull-stripping, spatial normalization, tissue segmentation, and surface reconstruction. (2) Functional data processing: slice timing correction, motion correction, coregistration to anatomical images, spatial normalization (applying the transformation derived from T1-weighted image registration to standard space onto the functional images), and component-based noise correction. To reduce the potential impact of tumor mass on spatial normalization, we employed lesion-masked registration: tumor regions defined by manual tumor masks were excluded from the cost function during registration. This approach helps minimize anatomical distortion caused by large tumors. (3) Nuisance regression included six head motion parameters (three translations and three rotations) along with their first-order derivatives. Physiological noise removal was performed using CompCor, extracting noise signals from white matter and cerebrospinal fluid. (4) Band-pass filtering (0.01 to 0.08 Hz) and linear detrending were applied to remove low-frequency drifts and high-frequency noise. (5) Spatial smoothing using a Gaussian kernel with a full width at half maximum (FWHM) of 6 mm. (6) Quality assurance included visual inspection of structural-functional alignment, spatial normalization accuracy, and noise removal efficiency.

**1.3. Brain Network Features**

The meanings of the various network features are as follows:

1) **Characteristic Path Length:** The average shortest path length between all pairs of nodes in the network, representing the efficiency of information transfer.
2) **Clustering Coefficient:** Indicates the degree to which nodes in a graph tend to cluster together, reflecting the network's local connectivity.
3) **Local Efficiency:** The average efficiency of the subgraphs composed of the nearest neighbors of each node, reflecting the fault tolerance of the network.
4) **Global Efficiency:** The average inverse shortest path length in the network, indicating the efficiency of parallel information transfer.

**1.4. Tumor Location and RIFR**

To examine whether tumor location affects RIFR, we implemented two analytical approaches. (1) Distance-Based Analysis. We calculated the shortest Euclidean distance from the tumor’s geometric center to each of the Yeo 17 brain networks and performed Spearman correlation analysis between this distance and the corresponding intratumoral RIFR values. (2) Overlap-Based Classification: Using the Yeo 17 atlas in MNI 152 space, we classified tumors into overlapping and non-overlapping groups for each network. The overlapping group included tumors intersecting with a given network, while the non-overlapping group consisted of tumors outside that network. We then compared RIFR between these two groups to assess whether tumor-network overlap influenced functional preservation within the tumor.

**2. Supplementary Results**

**2.1. Robustness of RIFR and RPTR Across Parcellation Schemes and FC Thresholds**

To evaluate the robustness of RIFR and RPTR, we performed additional analyses using multiple parcellation schemes and functional connectivity thresholds. Using the Yeo 7-network atlas for functional assignment, graph-theoretical metrics were extracted based on three different brain atlases with varying spatial resolutions, including AAL, Schaefer-100, and Schaefer-300 atlases. Analyses were conducted under FC thresholds of 0.3, 0.5, and 0.7. Across all combinations of atlases and thresholds, the relationships between RIFR/RPTR and network metrics showed high consistency with the main results. Although some associations did not reach statistical significance under lower thresholds or coarser parcellations, the overall trends remained stable.

Furthermore, we repeated the primary analyses shown in Figure 5 under the same conditions. The results demonstrated consistent associations between RIFR and pathological features, clinical manifestations, structural alterations, network properties, and functional impairments (Supplementary Figure S15).

These findings confirm that RIFR and RPTR are robust across different methodological choices, supporting their generalizability and reproducibility.

**2.2. Baseline Survival Prediction Model Using Conventional Clinical Variables**

To evaluate the incremental predictive value of tumor-related functional network features (RIFR, RPTR, VFR, VTR), we first constructed baseline survival prediction models using only conventional clinical variables. These included age, sex, tumor volume, and WHO grade. Both support vector machine (SVM) and logistic regression classifiers were applied. The model performance was evaluated using receiver operating characteristic (ROC) curve analysis and area under the curve (AUC) metrics. WHO grade demonstrated the highest predictive power (AUC = 0.763), followed by age (AUC = 0.664), tumor volume (AUC = 0.634), and sex (AUC = 0.618) (Supplementary Figure 16A). We then trained machine learning models using a combination of these four variables. The support vector machine (SVM) model achieved an AUC of 0.79, while the logistic regression model reached an AUC of 0.80 (Supplementary Figure 16B). These results serve as a reference baseline for evaluating the added predictive value of functional network features.

**Supplementary Figure S1**

**Supplementary Figure S1. Baseline characteristics of glioma patients pie chart.** This pie chart provides a visual distribution of baseline characteristics among the 306 glioma patients included in the study. The data presented includes gender distribution, WHO grade, molecular subtypes, IDH mutation status, EGFR amplification, 1p/19q codeletion, Ki-67 expression, TP53 mutation status, MGMT promoter methylation, TERT promoter mutation, ATRX, mutation, preoperative KPS, presence of epilepsy, cognitive impairments, and language function.

**Supplementary Figure S2**

**Supplementary Figure S2. Spearman correlation between the shortest Euclidean distance from the tumor geometric center to the Yeo 17 brain networks and corresponding RIFR values.**

**Supplementary Figure S3**

**Supplementary Figure S3. Violin plot comparing RIFR values in tumors overlapping vs. non-overlapping with each of the 17 Yeo brain networks.** N1: Visual A; N2: Visual B; N3: Somatomotor A; N4: Somatomotor B; N5: Dorsal Attention A; N6: Dorsal Attention B; N7: Salience/Ventral Attention A; N8: Salience/Ventral Attention B; N9: Limbic A; N10: Limbic B; N11: Control A; N12: Control B; N13: Control C; N: Default A; N15: Default B; N16: Default C; and N17: Temporal Parietal. ^***^*P* < 0.001.

**Supplementary Figure S4**

**Supplementary Figure S4. A schematic diagram of direct cortical stimulation-induced finger movement.**

**Supplementary Figure S5**

**Supplementary Figure S5. Effects of functional connectivity thresholds on RIFR with clinical and pathological features (Yeo 17 atlas). (A)** WHO grade; **(B)** IDH mutation status; **(C)** EGFR expression; **(D)** 1p/19q codeletion; **(E)** Ki-67 expression; **(F)** TP53 mutation; **(G)** MGMT methylation; **(H)** TERT promoter mutation; **(I)** ATRX mutation; **(J)** Preoperative KPS scores; **(K)** Preoperative epilepsy; **(L)** Tumor volume. ^*^*P* < 0.05, ^**^*P* < 0.01.

**Supplementary Figure S6**

**Supplementary Figure S6. Effects of functional connectivity thresholds on VFR with clinical and pathological features (Yeo 17 atlas). (A)** WHO grade; **(B)** IDH mutation status; **(C)** EGFR expression; **(D)** 1p/19q codeletion; **(E)** Ki-67 expression; **(F)** TP53 mutation; **(G)** MGMT methylation; **(H)** TERT promoter mutation; **(I)** ATRX mutation; **(J)** Preoperative KPS scores; **(K)** Preoperative epilepsy; **(L)** Tumor volume. ^*^*P* < 0.05, ^**^*P* < 0.01.

**Supplementary Figure S7**

**Supplementary Figure S7. Effects of functional connectivity thresholds on RPTR with clinical and pathological features (Yeo 17 atlas). (A)** WHO grade; **(B)** IDH mutation status; **(C)** EGFR expression; **(D)** 1p/19q codeletion; **(E)** Ki-67 expression; **(F)** TP53 mutation; **(G)** MGMT methylation; **(H)** TERT promoter mutation; **(I)** ATRX mutation; **(J)** Preoperative KPS scores; **(K)** Preoperative epilepsy; **(L)** Tumor volume. ^*^*P* < 0.05, ^**^*P* < 0.01.

**Supplementary Figure S8**

**Supplementary Figure S8. Effects of functional connectivity thresholds on VTR with clinical and pathological features (Yeo 17 atlas). (A)** WHO grade; **(B)** IDH mutation status; **(C)** EGFR expression; **(D)** 1p/19q codeletion; **(E)** Ki-67 expression; **(F)** TP53 mutation; **(G)** MGMT methylation; **(H)** TERT promoter mutation; **(I)** ATRX mutation; **(J)** Preoperative KPS scores; **(K)** Preoperative epilepsy; **(L)** Tumor volume. ^*^*P* < 0.05, ^**^*P* < 0.01.

**Supplementary Figure S9**

**Supplementary Figure S9. Correlation of intratumoral functional region proportion with patient age, tumor volume, and tumor location. (A)** Scatter plot of correlation between RIFR and age. **(B)** Scatter plot of correlation between RIFR and tumor volume. Probabilistic maps of tumor distribution in patients with high RIFR **(C)** and low RIFR **(D)**. The color range (from blue to red) indicates low to high probabilities (in percent) of tumor occurrence at each voxel.

**Supplementary Figure S10**

**Supplementary Figure S10.** Structural correlates of peritumoral functional network features. **(A)** Comparison of cortical thickness between ipsilesional and contralesional hemispheres in low and high RPTR groups. **(B, C)** Comparison of FA and MD across low RPTR, high RPTR, and healthy controls (HCs) at the whole-brain level and in tumor-involved white matter tracts (CST, AF, SLF). ^*^*P* < 0.05, ^**^*P* < 0.01, ^***^*P* < 0.001.

**Supplementary Figure S11**

**Supplementary Figure S11. Effects of language impairment on tumor-associated RIFR in the language network across different FC thresholds.** ^*^*P* < 0.05, ^**^*P* < 0.01.

**Supplementary Figure S12**

**Supplementary Figure S12. Correlations between RPTR and various graph theory properties and brain function impairments. (A-D)** Spearman correlation scatter plots explore the relationships between the RPTR and global brain network attributes including characteristic path length (A), clustering coefficient (B), local efficiency (C), and global efficiency (D). **(E, F)** Violin plots assess the differences in RPTR among patients with varying degrees of cognitive impairment (E) and the presence or absence of language deficits (F). ^**^*P* < 0.01.

**Supplementary Figure S13**

**Supplementary Figure S13. Robustness of RIFR–network associations across parcellation schemes (Yeo 7 atlas).** Correlation analyses between RIFR and network metrics using AAL (A-D), Schaefer-100 (E-H), and Schaefer-300 (I-L) atlases under different FC thresholds.

**Supplementary Figure S14**

**Supplementary Figure S14. Robustness of RPTR–network associations across parcellation schemes (Yeo 7 atlas).** Correlation analyses between RPTR and network metrics using AAL (A-D), Schaefer-100 (E-H), and Schaefer-300 (I-L) atlases under different FC thresholds.

**Supplementary Figure S15**

**Supplementary Figure S15. Reproducibility of main findings under Yeo 7 atlas and different FC thresholds. (A-D)** Violin plots illustrate the differences in RIFR across varying pathological grades (A), IDH mutation status (B), EGFR expression levels (C), 1p/19q codeletion (D). **(E, F)** Violin plots illustrate the differences in VFR across varying preoperative KPS scores (E) and the presence or absence of epilepsy (F). **(G)** Comparison of cortical thickness between ipsilesional and contralesional hemispheres in low and high RIFR groups. **(H, I)** Comparison of FA and MD across low RIFR, high RIFR, and healthy controls (HCs) at the whole-brain level. **(J-M)** Spearman correlation scatter plots explore the relationships between the RIFR and brain network properties including characteristic path length (J), clustering coefficient (K), local efficiency (L), and global efficiency (M). **(N, O)** Violin plots assess the differences in RIFR among patients with varying degrees of cognitive impairment (N, n = 79) and the presence or absence of language deficits (O, n = 85). Statistical significance is indicated by asterisks: ^*^*P* < 0.05, ^**^*P* < 0.01, ^***^*P* < 0.001, illustrating the levels of correlation and difference across the explored variables.

**Supplementary Figure S16**

**Supplementary Figure S16. Survival prediction model based on conventional clinical variables. (A)** ROC curves for single clinical predictors of 2-year survival, including WHO grade, age, tumor volume, and sex. **(B)** ROC curves for combined clinical model using SVM and logistic regression.

**Supplementary Figure S17**

**Supplementary Figure S17. Changes in intratumoral and peritumoral network metrics across different functional connectivity thresholds.** **(A)** Trend graph showing the decrease in RIFR and RPTR as functional connectivity thresholds increase. **(B)** Trend graph illustrating the reduction in VFR and VTR with increasing functional connectivity thresholds. FC, functional connectivity; VFR, the volume of the functional regions in core tumors; VTR, the volume of the tumoral regions in PSA area; RIFR, the ratio of the intratumoral functional regions to tumor volume; RPTR, the ratio of the peritumoral tumoral regions to PSA volume.
